# Methylation recognition protein YTH N6-methyladenosine RNA binding protein 1 (YTHDF1) regulates the proliferation, migration and invasion of osteosarcoma by regulating m6A level of CCR4-NOT transcription complex subunit 7 (CNOT7)

**DOI:** 10.1080/21655979.2022.2037381

**Published:** 2022-02-14

**Authors:** Kang Wei, Yi Gao, Bin Wang, Yu-Xing Qu

**Affiliations:** The First Department of Orthopadics, Changzhou Traditional Chinese Medical Hospital, Changzhou, China

**Keywords:** Osteosarcoma, YTH N6-methyladenosine RNA binding protein 1 (YTHDF1), CCR4-NOT transcription complex subunit 7 (CNOT7), methylated modification, malignant progression

## Abstract

N6-methyladenosine (m6A) is one of the most significant modifications in human mRNAs. Emerging evidence indicates that m6A participates in the initiation and development of malignant tumors. Nevertheless, the biological roles and mechanism of m6A in osteosarcoma (OS) remain unclear. The purpose of this study was to investigate the role and mechanism of the methylation recognition protein-YTH N6-methyladenosine RNA binding protein 1 (YTHDF1) in OS. The YTHDF1 expression in OS was detected by qRT-PCR and Western blot assay. M6A quantification was utilized to measure the methylation level of OS. Cell counting kit-8 (CCK8), 5-Ethynyl-2’-deoxyuridine (EdU) assay and transwell experiments were conducted to confirm the biological effects of YTHDF1 on OS cells. The bioinformatics websites and in vitro assays were conducted to analyze the downstream targets of YTHDF1 was upregulated in OS tissues at mRNA and protein level. The results showed that the expression level of YTHDF1 might be closely associated with the poor prognosis for OS patients. Inhibition of YTHDF1 could suppress the proliferation, migration and invasion of the OS cells. Moreover, we found that CCR4-NOT transcription complex subunit 7 (CNOT7) might be the potential target of YTHDF1, which was upregulated in OS tissues. YTHDF1 could recognize the m6A sites of CONT7 and promote its expression in an m6A manner. Moreover, methyltransferase-like 3 (METTL3) could promote the m6A level of CONT7. YTHDF1 was upregulated in OS and could promote cell proliferation, migration and invasion. The METTL3-CONT7-YTHDF1 regulatory axis might be the potential target for the prognosis and therapy of OS.

## GRAPHICAL ABSTRACT

**Figure:**
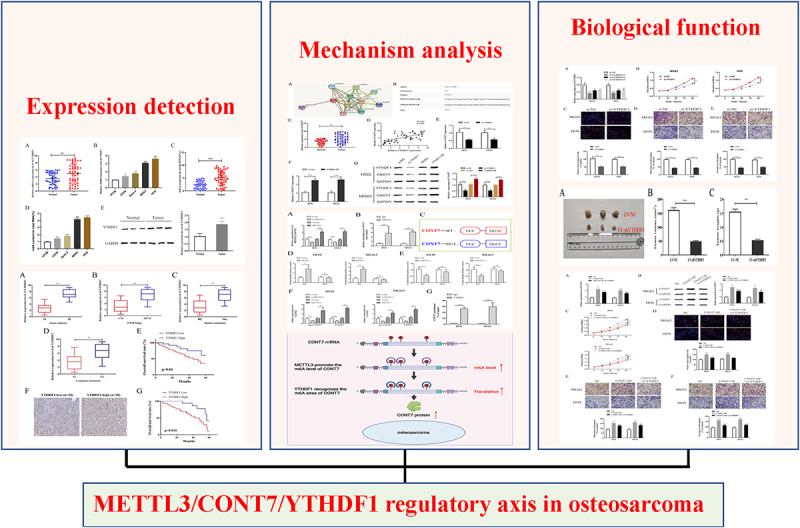

## Introduction

Osteosarcoma (OS) is a type of primary malignant bone tumor that derives from bone mesenchymal cells, with a higher incidence in children and adolescents. Since it betides more in blood-rich metaphysis [1], the 5-year survival rate of OS remains only 60%~70%[2]. About 18% of OS could metastase at an early stage, most of which occur in lungs [3]. A variety of clinical and genetic factors may be involved in the malignant development of OS [4]. Although surgical treatment and multi-drug chemotherapy have made great progress in recent years, the survival rate of OS patients remains unsatisfactory [5,6]. Therefore, it is particularly essential to explore new therapeutic targets and diagnostic biomarker of OS.

N6-methyladenosine (m6A) methylation of RNA is defined as the methylation of the 6th nitrogen atom of RNA adenine (A) base under the action of methyltransferase. The phenomenon of RNA m6A methylation was discovered as early as the 1970s [7]. With the development of high-throughput sequencing methods for m6A at the whole transcriptome level, researches on the specific molecular functions of m6A modification have developed rapidly [8]. It has been observed that if the m6A modification occurs in the 5ʹuntranslated region, it may promote the 5ʹcap end-independent protein translation of the transcript. If the m6A modification only occurs in 3ʹuntranslated region, it may bind to YTH N6-methyladenosine RNA binding protein 1 (YTHDF1) to further recruit the eukaryotictranslation initiation factor 3 (eIF3) and promote the 5’ cap end and 3ʹpoly tail end to form the cyclic structure, thus promoting the 5’ cap end-dependent protein translation. If the m6A modification occurs in the sequence coding region or 3’ untranslated regions (3ʹUTR), it may bind to YTHDF2 to further shorten the RNA 3ʹpoly tail end and promote mRNA degradation [9,10]. More and more studies have revealed that m6A methylation modification exerts a significant biological function in human malignant tumors. For instances, METTL3 may promote the proliferation and migration of pancreatic cancer cells [11]. FTO alpha-ketoglutarate dependent dioxygenase (FTO) can regulate the mRNA level of homeobox B13 (HOXB13) and promote the metastasis of endometrial cancer via modulating the WNT signaling pathway [12]. Methyltransferase-like 14 (METTL14) can suppress the growth and metastasis of colorectal cancer (CRC) via repressing X inactive specific transcript (XIST) [13]. However, YTHDF1 has not been fully studied in OS, and needs to be further explored.

In this study, we confirmed that YTHDF1 expression was elevated in OS. Overexpression of YTHDF1 could promote cell proliferation, migration and invasion. YTHDF1 could regulate the expression of CONT7 through the m6A dependent manner. Hence, the purpose of this study was to verify the biological function and mechanism of YTHDF1 and CONT7 in OS. The METTL3/CONT7/YTHDF1 regulatory axis might be the potential pathogenesis and therapeutic target of OS.

## Methods

### Sample collection

The OS tissue specimens and chondroma tissue specimens were collected from the surgical sample archives of Changzhou Traditional Chinese Medical Hospital. All specimens were frozen in liquid nitrogen after surgical isolation and stored in −80°C. These specimens were obtained with informed consent from the patients receiving surgery and approved by the ethics committee of Changzhou Traditional Chinese Medical Hospital.

### Cell culture

The normal human osteoblast (hFOB) and OS cell lines (MG63, U2OS, HOS, Saos-2) were obtained from the ATCC (Manassas, USA). All the OS cells were maintained in the DMEM Medium (Invitrogen; USA) supplemented with 10% FBS (Gibco) and 1% penicillin/streptomycin. While hFOB cells were cultured in DMEM medium/Nutrient Mixture F-12 (DMEM/F-12) containing 10% FBS and 0.3 mg/ml G418 (Invitrogen, USA). All the cells were stored in the humidified atmosphere with 5%CO_2_ at 37°C.

### Cell transfection

Small interfering RNAs (siRNAs) for YTHDF1 and CNOT7 were constructed by GenePharma Co., Ltd (Shanghai, China). Overexpressed plasmids (OE) of YTHDF1 and CNOT7 were obtained from RIBOBIO (Guangzhou, China). With 70%-80% of cell adherence, the cell transfection was conducted by utilizing Lipofectamine 3000 reagent (Life, USA) according to the manufacturer’s instructions [14]. The transfected cells were stored in the humidified incubator with 5%CO_2_ at 37°C. The concentration of siRNAs was 50 nM and the OE was 40 μg/ml.

The construct the stable MG-63 cell line down-expressing YTHDF1, the lentivirus-shYTHDF1 were obtained from GeneChem (Shanghai, China). The YTHDF1 sequences were synthesized and cloned into pcDNA3.1 vector and the HEK-293 T cells were utilized to generate packaging plasmids. After infection, the MG-63 cells were selected with puromycin for 2 weeks.

### Quantitative reverse transcription polymerase reaction (qRT-PCR)

The TRIzol reagent (Invitrogen, NY) was utilized to isolate the total RNAs from tissues and cells according to the manufacturer’s instructions. Then the RNA was reversely transcribed into complementary DNAs (cDNAs) through using PrimeScript RT kit (Takara Biotechnology Inc.). The SYBR Green (Takara Biotechnology Inc.) regent was utilized to conduct qRT-PCR assay. The reaction conditions were listed as follows: 95°C for 10 minutes for 1 cycle, denaturation at 95°C for 30 seconds, annealing at 56°C for 1 minutes, extension at 72°C for 30 seconds for a total of 40 cycles. The relative expression level was measured utilizing the 2^−ΔΔCT^ method [15]. GAPDH was used as internal references. The primer sequences were showed as below:

YTHDF1:F: 5’- ACCTGTCCAGCTATTACCCG-3’

R: 5’- TGGTGAGGTATGGAATCGGAG-3’

CNOT7: F: 5’- ATGCCAGCGGCAACTGTAG-3’

R: 5’- TCGGTGTCCATAGCAACGTAA-3’

METTL3: F: 5’- TTGTCTCCAACCTTCCGTAGT-3’

R: 5’- CCAGATCAGAGAGGTGGTGTAG-3’

GAPDH: F: 5’-CTCGCTTCGGCAGCACA-3’

R: 5’-ACGCTTCACGAATTTGCGT-3’

### Immunohistochemistry (IHC)

IHC analysis was conducted utilizing anti-YTHDF1 (ab230330, Abcam) as pervious described [16]. The OS tissues were heated in citrate buffer at 98°C for 20 min and cooled to RT for antigen retrieval. Then the specimens were cultured with YTHDF1 antibody at 4°C overnight and followed by incubating with secondary antibody at room temperature for 2 h. The tissues were further stained with streptavidin-biotin-peroxidase reagents and fixed on gelatin-coated glass slides. Then the average number of YTHDF-positive cells were measured.

### RNA m6A quantification

The m6A RNA methylation assay kit (Abcam) was utilized to conduct m6A content analysis of total RNAs according to manufacturer’s protocol [17]. Briefly, we coated 200 ng of RNA on a 96-well plate and then added the corresponding antibody and enhancer solution. The detected signal in each well was colorimetrically quantified by reading the absorbance at 450 nm with a microplate reader. Finally, we calculated the m6A level by the given formula in the manufacturer’s instructions.

### m6A immunoprecipitation (MeRIP) assay

Magna MeRIP m6A Kit (Millipore, USA) was used to conduct the MeRIP assay in accordance with the manufacturer’s instructions [18]. Anti-m6A body (MABE1006, Merck Millipore), anti-METTL3 body (ab195352, Abcam, USA) and anti-DDDK tag body ((ab205606, Abcam, USA) were applied for MeRIP detection. After washing with IP buffer, we eluted and subject RNA to the ethanol precipitation. Then, qRT-PCR was conducted to measure the enrichment of m6A containing mRNA.

### CCK8 experiment

The Cell Counting Kit-8 (CCK-8, Dojindo, Japan) was utilized for the detection of the proliferative ability of MG63 and HOS cells according to the manufacturer’s instructions [19]. Transfected MG63 and HOS cells were placed into the 96-well plate at 5 × 10^3^ cells/well. At different time points (0, 24, 48, 72 and 96 h, respectively), 10 μl of CCK-8 reagent (Dojindo, Japan) was seeded into each well, and incubated for another 2 h away at dark. Finally, the absorbance was recorded at 450 nm utilizing a microplate reader (Bio Tek, USA).

### Dual-luciferase reporter gene assay

Mutant-type and wild-type sequences of the 3’-UTR of CONT7 were constructed by GenePharma Co., Ltd (Shanghai, China). In brife, the firefly luciferase plasmids of mutant-type or wild-type m6A motifs of CONT7 3′UTR, METTL3 shRNAs, or GV657-flag and Ranilla plasmids were together transferred to OS cells. After co-culture for 48 h, the luciferase activity in transfected OS cells was measured using the dual-luciferase reporter experiment (Promega, USA) [20].

### 5-Ethynyl-2’-deoxyuridine (EdU) experiment

The EdU kit (Invitrogen, USA) was applied for the detection of the proliferative ability of MG63 and HOS cells according to the manufacturer’s instructions [21]. The transfected OS cells were placed in 96-well plates at the density of 5 × 10^3^/well. After adding 10 μl of EdU reagent into each well, cells were incubated for 3 h. Then the cells were fixed with 4% formaldehyde at room temperature for 20 min, washed off using PBS for three times, and the cells were subsequently incubated with 0.5% Triton X-100 for 20 min. Finally, the cells were stained with DAPI and photographed by the fluorescence microscope (Olympus, Japan).

### Transwell experiment

The transwell assay was conducted by utilizing transwell chambers, as described previously [22]. Following 24 h of cell transfection, cell digestion, collection, and counting were conducted; FBS-free RPMI-1640 medium was used for cell re-suspension, and cells were resuspended at a density of 2 × 10^5^/mL. The upper transwell chamber (Corning, USA) was seeded with 200 μL of above cell suspension, while the lower chamber was filled with DMEM medium consist with 10% FBS for 24 h of conventional culture in the cell incubator prior to removal. Then the cells were washed with PBS for three times and fixed in 4% paraformaldehyde for 10 min, followed by staining with crystal violet for 20 min. The OS cells were dried at room temperature and observed, counted, and photographed under an inverted microscope. No substrate gel was required for the migration experiment, whereas substrate gel was pre-distributed in the upper chamber for the invasion experiment. The remaining steps were completed in an identical fashion.

### In vivo xenograft assay

BALB/c nude mice were housed in the SPF grade animal house with a 12 h light/dark cycle and constant temperature. Then the mice were divided into two groups with three mice in each group and their flank was subcutaneously injected with 1 × 10^7^ OS cells. 28 days following subcutaneous injection, the mice were sacrificed through carbon dioxide euthanasia (30%/min) to obtain tumor weight and volume measurements. The study protocol was approved by the Ethics Committee of the Changzhou Traditional Chinese Medical Hospital, and animal experiments were conducted by following the Guide for the Care and Use of Laboratory Animals (NIH, USA).

### Western blotting

Western blotting was performed as previously described [23]. After digestion and suspension, the transfected MG63 and HOS cells were added into a 6-well plate at the cell density of 2 × 10^6^/well; and the protein expression was measured after culturing for 24 h. The protein lysis buffer (RIPA: PMSF = 100:1) was used to extract total proteins, while the BCA method was used to measure total proteins. About 60 µg protein from each group was subjected to electrophoresis and then transferred into the PVDF membrane. Subsequently, the membrane was blocked at room temperature for 1 h and incubated with the primary antibody at 4°C for overnight. The following day, horseradish peroxidase labeled secondary antibody was added. Color development ensued following addition of chromogenic solution; photographs were taken for analysis 1 h post incubation. The antibodies used were as follows: YTHDF1 (ab220162, 1/1000, abcam), CONT7 (ab195587, 1/1000, abcam), GAPDH (60,004-1-Ig, 1/1000, proteintech), and the secondary antibodies peroxidase-conjugated with the anti-rabbit and anti-mouse (Santa Cruz Biotechnology).

### Statistical analysis

The data and figures were conducted by utilizing GraphPad Prism 7.0 software. All data were revealed as the mean ± standard error from three independent experiments. The Student’s t tests and one-way ANOVA were utilized for two groups and multiple comparisons, respectively. Fisher’s exact test was analyzed to confirm the correlation between YTHDF1 expression level and OS clinicopathological features. Kaplan-Meier method was utilized to generate the survival curves. P values < 0.05 were considered statistically significant.

## Results

In this study, we demonstrated that the methylation recognition protein YTHDF1 could modulate the expression of CONT7 via an m6A dependent manner in OS. Hence, we aimed to identify the biological function and mechanism of the METTL3-CONT7-YTHDF1 regulatory axis in OS. The results showed that YTHDF1 was up-regulated in OS tissues and cell lines. Inhibition of YTHDF1 could significantly suppress the proliferative, migrative and invasive abilities of OS cells. Besides, the mRNA expression of CONT7 was rise in OS tissues and the m6A level could be promoted by METTL3. Moreover, YTHDF1 could promote CONT7 translation in am m6A dependent manner. Overall, this work revealed the function and mechanism of the METTL3-CONT7-YTHDF1 regulatory axis in OS, which might be the novel target for OS diagnosis and therapy.

### YTHDF1 was highly expressed in OS

**Figure 1.**
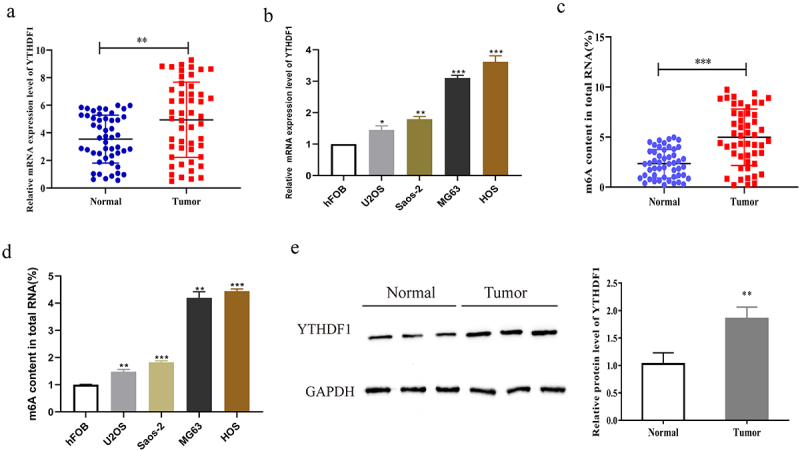
YTHDF1 was highly expressed in OS. a. The expression level of YTHDF1 in OS tissues was detected by qRT-PCR. b. The expression level of YTHDF1 in OS cell lines was detected by qRT-PCR. c. M6A level of OS tissues and normal controls were detected by RNA m6A quantification assay. d. M6A level of OS cell lines were detected by RNA m6A quantification assay. E. Western blot was used to detect the protein expression of YTHDF1 in OS tissues (*P < 0.05; **P < 0.01; ***P < 0.001).

Firstly, qRT-PCR was carried out to verify the expression level of YTHDF1 in OS tissues and normal controls. As shown in Figure 1(a), YTHDF1 expression was significantly higher in OS tissues. Moreover, we further measured the expression level of YTHDF1 in osteoblasts and OS cell lines. As indicated in Figure 1(b), the mRNA level of YTHDF1 in OS cell line was notably higher than that in osteoblasts. Subsequently, m6A detection was performed to further analyze the m6A methylation level of OS tissues. The results verified that the m6A level in OS tissues was remarkably increased (Figure 1(c)). We also detected the m6A level in normal osteoblasts and OS cell lines, which demonstrated that the m6A expression level in OS cell lines was notably higher than that in normal cells (Figure 1(d)). Moreover, the protein level of YTHDF1 in OS tumor tissues and adjacent tissues was measured through Western blot experiment (Figure 1(e)). The results indicated that the protein level of YTHDF1 in OS tumor tissues was also increased. These results showed that YTHDF1 might act as an oncogene in OS.

### YTHDF1 expression was associated with the prognosis of OS

**Figure 2.**
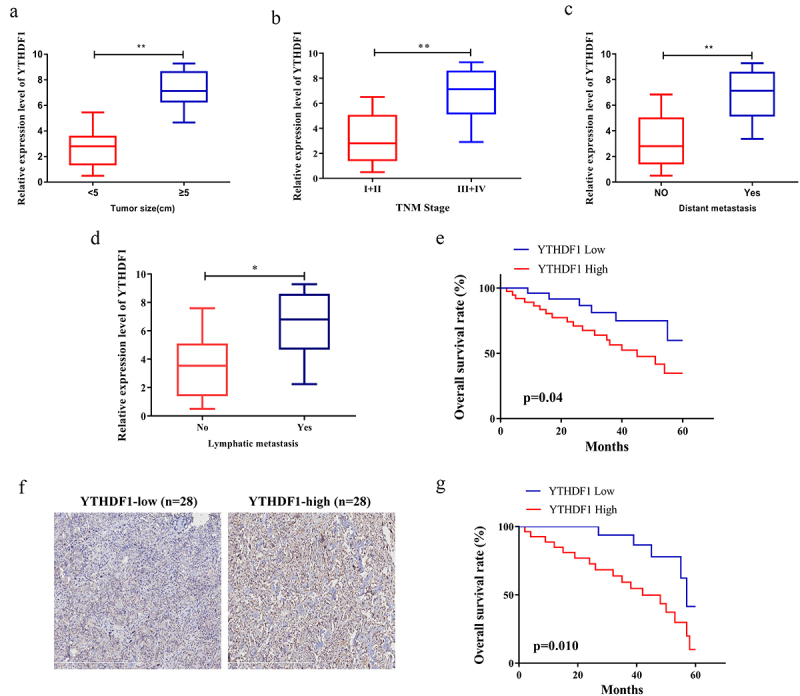
The expression level of YTHDF1 was correlated with the clinical characteristics of OS patients. a. The expression level of YTHDF1 in different OS tumor diameters. b. The expression level of YTHDF1 in different OS TNF stage. c. The relationship between the expression level of YTHDF1 and the occurrence of distant metastasis in OS patients. d. The correlation between the expression level of YTHDF1 and the occurrence of lymphatic metastasis in OS patients. e. The relationship between the expression level of YTHDF1 and the overall survival rate of OS patients. f. Immunohistochemical staining showing the expression of YTHDF1 in tumor tissues of OS patients. g. The 5-year overall survival rate of patients with OS. The statistical analysis was performed using Log-rank (Mantel-Cox) test. (**P < 0.01).

The analysis performed to explore the influence of YTHDF1 expression on clinical characteristics revealed that YTHDF1 expression in OS patients with a tumor diameter < 5 cm was significantly lower than in patients with a tumor diameter ≥ 5 cm (Figure 2(a)). Meanwhile, OS patients at lower TNM stage (I+ II) also showed notably lower YTHDF1 levels in comparison with patients at a higher stage (III+IV) (Figure 2(b)). In addition, we analyzed and found that the OS patients with higher expression levels of YTHDF1 were susceptible to distant metastasis and lymphatic metastasis (Figure 2(c,d)). We also found that in comparison with the OS patients with a lower expression of YTHDF1, patients with a higher expression of YTHDF1 had a significantly higher total survival rate (Figure 2(e)). To further explore the clinical significance of YTHDF1 in OS, IHC stainings were conducted on the paraffin-embedded OS samples (n = 56) (Figure 2(f)). Kaplan-Meier analysis revealed that the overall survival (OS) rate of patients with lower YTHDF1 expression were markedly higher than those of patients with higher YTHDF1 expression (Figure 2(g)). YTHDF1 expression was subject to systemic analysis. As displayed in Table 1, higher expression of YTHDF1 was strongly associated with OS patients’ tumor diameter, TNM staging, and distant metastasis. Thus, the results indicated that high expression of YTHDF1 might result in a poor prognosis in OS patients.

**Table 1.** Relationship between YTHDF1 expression and the clinical pathological characteristics of OS patients (n = 50)

Clinic pathological features		NO. of cases	YTHDF1 expression		*p* – value
			Low (n = 25)	High (n = 25)	
Gender	Male	31	15	16	*P* > 0.05
	Female	19	10	9	
Age	≤20	35	17	18	*P* > 0.05
	> 20	15	8	7	
Tumor size	<5 cm	29	19	10	*P* < 0.01
	≥5 cm	21	6	15	
TNM stage	I/II	28	18	10	*P* < 0.01
	III/IV	22	7	15	
Lymph node metastasis	Negative	44	24	20	*P* > 0.05
	Positive	6	1	5	
Distant metastasis	No	34	22	12	*P* < 0.01
	Yes	16	3	13

### Inhibition of YTHDF1 could suppress the proliferation, migration and invasion of OS cells

**Figure 3.**
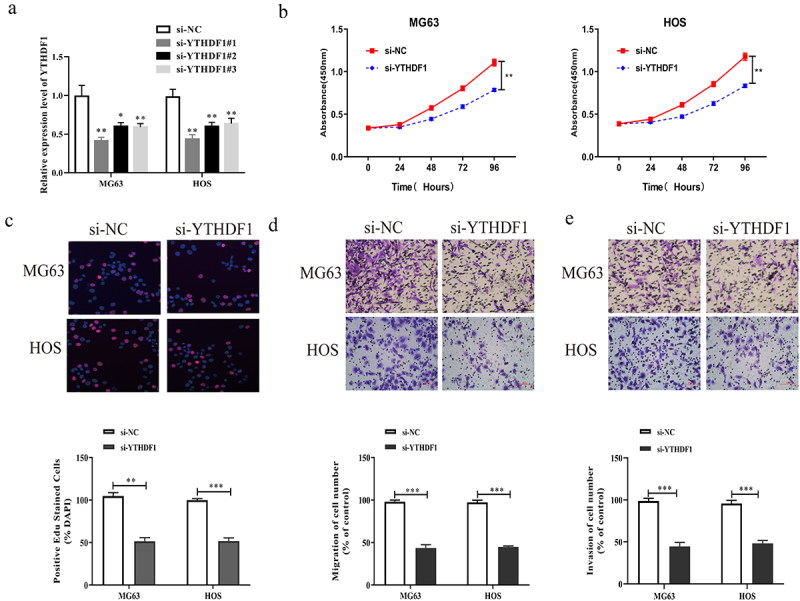
Down-regulation of YTHDF1 could inhibit the proliferation, migration and invasion of OS cells. a. The expression level of YTHDF1 in OS cells transfected with YTHDF1 siRNAs were detected by qRT-PCR. b. CCK8 assay was used to detect the absorbance of OS cells transfected with si-YTHDF1 at 450 nm. c. EdU assay was used to detect the positive rate of EdU in OS cells transfected with si-YTHDF1 (Magnification: 200X). d. The migration ability of OS cells was detected by transwell migration assay (Magnification: 200X). e. The invasion ability of OS cells was detected by transwell invasion assay (Magnification: 200X)(*P < 0.05; **P < 0.01; ***P < 0.001).

To investigate the influences of YTHDF1 on the malignant phenotype of OS cells, we down-regulated YTHDF1 expression level in MG63 and HOS cells using siRNAs, and we detected the interfering efficiency through qRT-PCR. As displayed in Figure 3(a), si-YTHDF1 transfected MG63 and HOS cells showed notably lower YTHDF1 expression than cells transfected with si-NC, whereas si-YTHDF1-1 transfected MG63 and HOS cells showed better interference efficiency. CCK8 experiments were carried out to detect the absorbance value at 450 nm for si-YTHDF1 transfected MG63 and HOS cells. The results revealed that, in comparison to si-NC, si-YTHDF1 transfected MG63 and HOS cells had a significantly lower absorbance value at 450 nm (Figure 3(b)). Meanwhile, EdU assay was utilized to further measure the proliferation of si-YTHDF1 transfected MG63 and HOS cells; the results indicated that si-YTHDF1 transfected MG63 and HOS cells showed notably lower Edu positive rate than si-NC transfected cells (Figure 3(c)). Subsequently, the migrative and invasive abilities of si-YTHDF1 transfected MG63 and HOS cells were detected by Transwell experiments. Moreover, si-YTHDF1 transfected MG63 and HOS cells showed significantly lower migration and invasion than cells transfected with si-NC (Figure 3(d,e)). These findings revealed that the repression of YTHDF1 suppressed the malignant phenotype of OS cells.

### Down regulation of YTHDF1 can inhibit the growth of OS tumor

**Figure 4.**
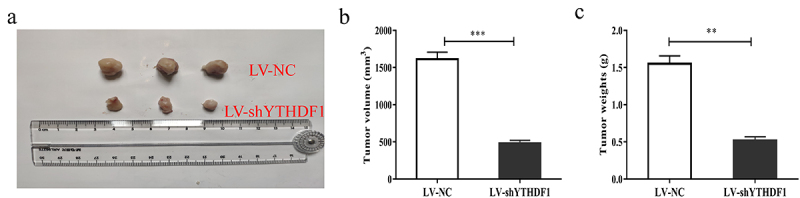
Down regulation of YTHDF1 could inhibit the growth of OS. A-C. Tumor volume and weight in nude mice injected with Lv-shYTHDF1(**P < 0.01; ***P < 0.001).

We investigated the effect of down regulation of YTHDF1 on OS tumor growth. As shown in Figure 4(a-c), after four weeks, the tumor growth volume and weight of nude mice injected with OS cells transfected with LV-shYTHDF1 were obvious smaller than those of NC. All above results suggested that down-regulation of YTHDF1 might inhibit OS tumor growth.

### YTHDF1 regulated the m6A level of CNOT7

**Figure 5.**
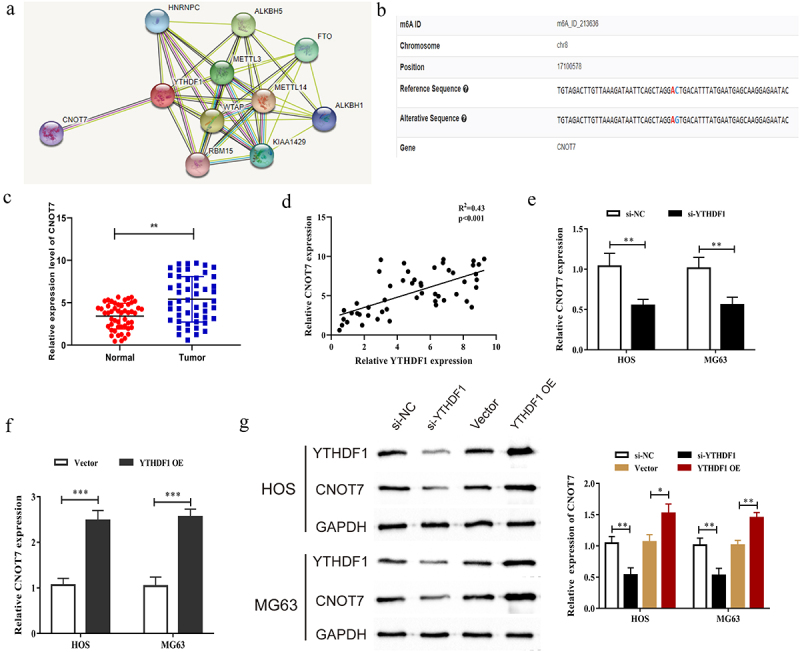
YTHDF1 could regulate the expression of CNOT7. a. Prediction of RNA binding proteins that may interact with YTHDF1 through bioinformatics website (https://cn.string-db.org). b. Prediction of CNOT7 methylation sites through bioinformatics websites (http://m6avar.renlab.org). c. Detected mRNA expression level of CNOT7 in OS tissues by qRT-PCR. d. Pearson method was used to analyze the correlation between the expression levels of YTHDF1 and CNOT7. e. The mRNA expression of CNOT77 in OS cells transfected with si-YTHDF1 was detected by qRT-PCR.f. The mRNA expression of CNOT77 in OS cells transfected with YTHDF1 OE was detected by qRT-PCR. g. The protein expression of CNOT7 in OS cells transfected with si-YTHDF1 or YTHDF1 OE was detected by Western blot (*P < 0.05; **P < 0.01; ***P < 0.001).

To further explore the molecular mechanism of YTHDF1 in OS, we predicted the downstream targets that might interact with YTHDF1 through STRING database (https://cn.string-db.org)to screen the potential targets of YTHDF1. The results showed that CNOT7 had the potential to co-express with YTHDF1 (Figure 5(a)). Then, we also found that CNOT7 exerted several methylation sites via bioinformatics analysis (http://m6avar.renlab.org) (Figure 5(b)). Moreover, we measured the mRNA expression of CNOT7 in OS tissues and found that the CNOT7 expression in OS tumor tissues was remarkedly increased (Figure 5(c)). We analyzed the correlation between expression level of YTHDF1 and CNOT7 using Pearson’s method. As displayed in Figure 5(d), their expression levels showed a significantly positive relationship between YTHDF1 and CONT7. We then detected the mRNA expression of CNOT7 in MG63 and HOS cells after the downregulation or overexpression of YTHDF1 through qRT-PCR experiments. The results verified that the mRNA expression of CNOT7 was decreased after the inhibition of YTHDF1, while increased after upregulating YTHDF1 in OS cells (Figure 5(e,f)). Subsequently, Western blot experiments identified that the protein expression of CNOT7 was remarkably reduced post the downregulation of YTHDF1, whereas the protein levels of CNOT7 were significantly increased after the upregulation of YTHDF1 (Figure 5(g)). All above results indicated that YTHDF1 might promote CONT7 in OS.

### METTL3 could regulate the m6A level of CONT7

**Figure 6.**
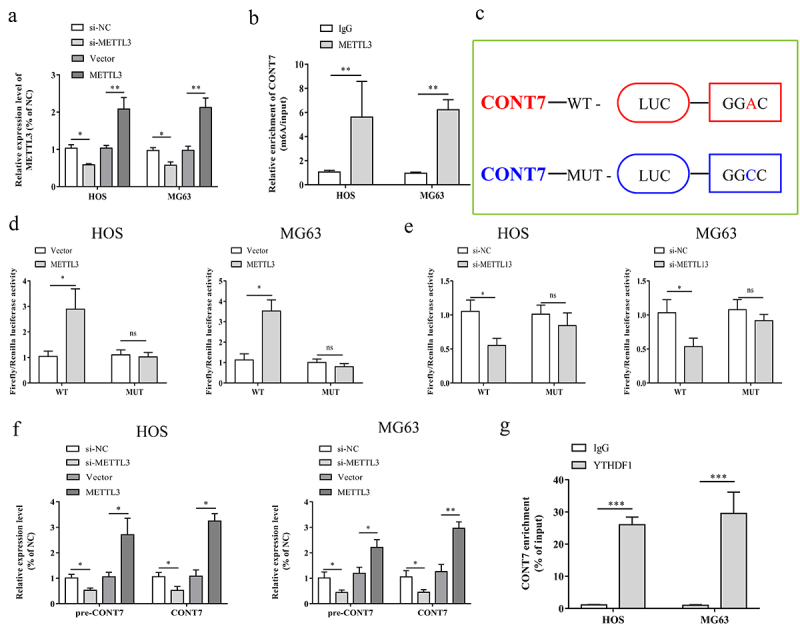
METTL3 overexpression promoted CONT7 mRNA stability via an m6A-YTHDF1-dependent pathway. a. The protein expression of METTL3 in OS cells transfected with si- METTL3 or METTL3 OE was detected by qRT-PCR. b. MeRIP-qPCR analysis was used to identify METTL3-mediated CONT7 m6A modification in HOS and MG63 cells. m6A modification of CONT7 was elevated upon METTL3 overexpression. c. Wild-type or m6A consensus sequence mutant CONT7 cDNA was fused with firely luciferse reporter. d-e. Mutation of m6A consensus sequences or METTL3 overexpression plasmid (or siRNA) promoted the transcription activity of CONT7 in HOS and MG63 cells. f. Precursor and mature mRNA of CONT7 in METTL3 overexpression or inhibition and normal controls in OS cells were detected by qRT-PCR. G. RIP-qPCR assay using YTHDF1-specifc antibody and IgG control antibody to measure the enrichment of YTHDF1 binding to CONT7 m6A modification sites.

Previous studies have showed that METTL3 expression is elevated in OS and could regulate the m6A level of other genes [16,24,25]. To identify the regulation of METTL3 on CONT7, we used siRNAs or overexpressing plasmid to decrease or elevate the expression of METTL3 in OS cells (Figure 6(a)). Then through the MeRIP-qPCR detection, we confirmed that upregulation of METTL3 in OS cells could promote the m6A level (Figure 6(b)). Moreover, we constructed wild-type or mutant CONT7 to explore the influences of m6A modification on CONT7 expression. The adenosine bases in the m6A consensus sequence (RRACH) were replaced by cytosine in the mutant form to cancel the m6A modification of CONT7 (Figure 6(c)). The results of dul-luciferase reporter gene assay indicated that the relative luciferase activity of CONT7 3’-UTR with wild-type m6A site was remarkedly decreased after METTL3 inhibition, while upregulation of METTL3 could promote the luciferase activity of CONT7 (Figure 6(d,e)). To further explore the molecular mechanism of the regulation of CONT7 expression by METTL3, we examined the expression level of CONT7 precursor (pre- CONT7) and mature (CONT7) mRNA in HOS and MG63 cells after overexpressing or knocking down METTL3. The results verified that METTL3 could promote the expression of pre- CONT7 and mature mRNA (Figure 6(f)). Additionally, the results of the RIP experiment confirmed that compared with the IgG control, the YTHDF1 specific antibody could lead to the enrichment of CONT7 mRNA (Figure 6(g)).

### Inhibition of YTHDF1 partially reversed the positive effect of upregulation of CONT7 on the malignant phenotype of OS cells

**Figure 7.**
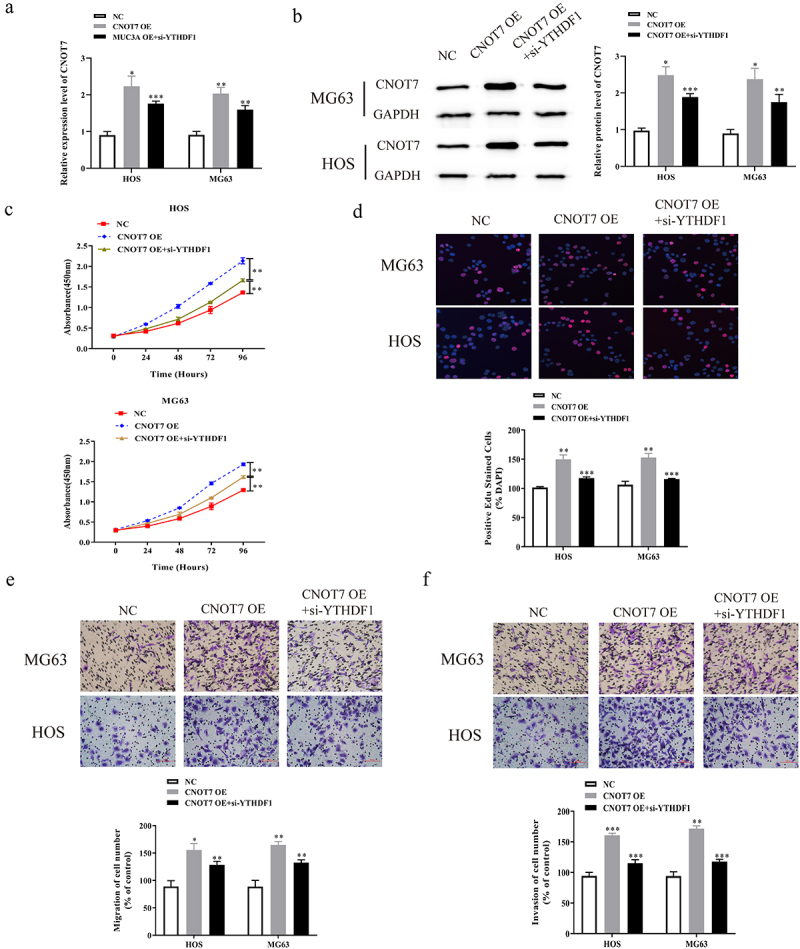
Down regulation of YTHDF1 partially reversed the promotion effect of high expression of CONT7 on the malignant phenotype of OS cells. a. mRNA expression of CNOT7 in OS cells co transfected with CNOT7 OE and si-YTHDF1 was detected by qRT-PCR. b. as used to detect the absorbance of OS cells co transfected with CNOT7 OE and si-YTHDF1 at 450 nm. c. EdU assay was used to detect the positive rate of EdU in OS cells co transfected with CNOT7 OE and si-YTHDF1 (Magnification: 200X). d. The migration ability of OS cells co transfected with CNOT7 OE and si-YTHDF1 was detected by Transwell migration assay (Magnification: 200X). e. The invasion ability of OS cells co transfected with CNOT7 OE and si-YTHDF1 was detected by Transwell invasion assay (Magnification: 200X) (*P < 0.05; **P < 0.01; ***P < 0.001).

To further explore the combined action of YTHDF1 and CNOT7 on the development of OS, we co-transfected CNOT7 overexpression plasmids and si-YTHDF1 in MG63 and HOS cells, and the transfection efficiency was measured using qRT-PCR. The findings showed that, after the co-transfection with CNOT7 OE and si-YTHDF1, the mRNA expression of CNOT7 was lower compared to MG63 and HOS cells transfected with CNOT7 OE, while remaining higher than the NC (Figure 7(a)). Subsequently, CCK8 experiments were conducted to detect the absorbance value of MG63 and HOS cells co-transfected with CNOT7 OE and si-YTHDF1 at 450 nm. The results showed that the downregulation of YTHDF1 partially reversed the promotion effects of CNOT7 on the absorbance values of MG63 and HOS cells at 450 nm (Figure 7(b)) Meanwhile, Edu experiments revealed that the downregulation of YTHDF1 partially reversed the promotion effects of high expression of CNOT7 on the EdU positive rates of OS cells (Figure 7(c)). Subsequently, Transwell migration experiments were conducted to detect the migration of MG63 and HOS cells co-transfected with CNOT7 OE and si-YTHDF1. We found that the downregulation of YTHDF1 partially reversed the positive effects of upregulation of CNOT7 on the migration of MG63 and HOS cells (Figure 7(d)). Meanwhile, Transwell invasion experiments uncovered that the downregulation of YTHDF1 partially reversed the promotion the effects of high expression of CNOT7 on MG63 and HOS cells (Figure 7(e)).

## Discussion

OS accounts for about 50% of all bone tumors. Adolescents aged 10 to 20 years are a high-risk population for developing OS. OS is prone to metastases, and most patients exhibit lung metastasis at the time of their first visit [26]. Studies have shown that the lung metastasis rate of OS patients at their first visit is as high as 20%, and this is one of the leading reasons for poor prognosis in OS patients [27]. Therefore, more novel targets are needed for earlier prognosis and treatment of OS. In the early stage of this study, we demonstrated that YTHDF1 expression was lower in OS tissues and cell lines. We speculated that YTHDF1 might function as an oncogene in OS. We verified this conjecture through qRT-PCR and Western blot experiments, and we analyzed the association between YTHDF1 expression level and clinical features of OS patients. We found that patients with low YTHDF1 expression level had a poorer prognosis and a lower total survival rate, indicating that YTHDF1 was possibly interrelated to poor prognosis of OS patients.

M6A is the most common mRNA modification in mammalian cells, accounting for 0.1–0.4% of all adenine residues [28]. M6A modification is crucial in a variety of cellular processes such as development, cell self-renewal, cell differentiation, DNA damage response, and cancer [29] In 1998, Imai et al. found the first YTH protein when screening TRA-2β (a component of the cleavage complex) interacting protein using the yeast two-hybrid method; this was named YT521-B [30]. The protein with YTH domain was subsequently named the YTH protein. Subsequently, BLAST comparison found that a region of 140 amino acids in the protein was highly conserved in its homologous protein, and this region was named the YTH (YT521-B homologs) domain [31]. The YTH domain protein family, as the earliest discovered and most important recognition protein family found for m6A modification, mainly includes two subtypes, YTHDF and YTHDC. YTHDF, primarily including YTHDF1, YTHDF2, and YTHDF3, which are typically located in the cytoplasm. YTHDF1 binds to the m6A site around the stop codon to increase the delivery of the mRNA transcription complex to promote translation initiation and protein synthesis in combination with translation initiation mechanisms [32]. Previous studies have indicated that YTHDF1 plays an important bio-function in human malignant tumors. For instance, Liu et al. found that YTHDF1 promoted the development of OC by promoting the transcription of EIF3C [33]. Pi et al. revealed that YTHDF1 promoted the development of gastric cancer by regulating the translation of FZD7 [34]; Zhao et al. indicated that high expression of YTHDF1 was strongly associated with a poor prognosis of liver cancer patients [35]. In this study, YTHDF1 expression in OS cells was downregulated using siRNA. CCK8, EdU, and transwell experiments uncovered that the proliferative, migrative, and invasive abilities of OS cells after the downregulation of YTHDF1 were lower compared to si-NC transfected control group. The results indicated that YTHDF1 might promote the malignant phenotype of OS cells as an oncogene.

CNOT7 is an important subunit of the eukaryote CCR4-NOT protein complex [36], and it may participate in the regulation of the transcription of multiple tumor microenvironment related proteins [37]. Previously, CNOT7 was found to exert specific bio-functions in breast cancer, ovarian cancer, colorectal cancer, and other malignant tumors [38–40]. This study serves as a preliminary discussion regarding the biological role of CNOT7 in OS, however, the role of CNOT7 in OS warrants further exploration. In this study, we used a bioinformatics website to predict whether the methylation site of CNOT7 is regulated by YTHDF1, and we made a series of analyses to verify this conjecture. We found that the expression levels of CNOT7 and YTHDF1 were significantly positively correlated, and that YTHDF1 might regulate CNOT7 expression through its impact on m6A levels. Moreover, we found that METTL3 could elevate the m6A level of CONT7 in OS cells. METTL3 could promote the expression of pre-CONT7 and CONT7. The RIP experiment verified that YTHDF1 could bind to CONT7. Combining the previously published articles and the biological characteristics of YTHDF1, we speculated that YTHDF1 might recognize the m6A sites of CONT7 and promote its transcription.

Overall, we showed the oncogenic role and the new epigenetic regulation METTL3-CONT7-YTHDF1 axis. Suppression of YTHDF1 could block proliferation, migration and invasion in OS cell lines and mouse xenograft model, which indicated that targeting YTHDF1 in cancer cells could be an effective therapeutic strategy. METTL3 could exert oncogene effects in OS cells through regulating the m6A level of CONT7, while YTHDF1 could recognize the m6A modification sites of CONT7 and promote the stability of CONY7. Taken together, our findings provided new light into the critical role of YTHDF1 in OS development, and indicated the novel significance of the molecular mechanism of m6A epi-transcriptomic modification in cancer research. The METTL3-CONT7-YTHDF1 axis might provide fresh insight for OS-targeted therapy.

Although our study provided the new clue to the molecular mechanism of OS, several limitations should also be noted. First, CONT7 was a potential downstream gene of YTHDF1 predicted by bioinformatics. The potential downstream gene of YTHDF1 can be studied by MERIP-sequence and qRT-PCR-sequence detection, which may make the research more novel. Second, we did not establish a metastasis model to explore the effect of YTHDF1 on tumor metastasis in vivo. Third, the role and mechanism of METTL3 in osteosarcoma were not further demonstrated in this study. Finally, we should collect more clinical specimens to investigate the potential translational potential of METTL3, CONT7, and YTHDF1 in OS diagnosis and prognostic assessment.

In future research, we will also analyze the Therapeutically Applicable Research To Generate Effective Treatments (TARGET) database and conduct corresponding basic experiments to explore the roles and potential molecular mechanisms of other m6A regulators in OS. According to the results, the complete signaling pathway of m6A modification in the occurrence and development of OS may be established, which can provide a theoretical basis for the early diagnosis, prognosis evaluation and targeted therapy of OS.

## Conclusion

YTHDF1 might act as an oncogene in OS via regulating the expression of CNOT7 in an m6A dependent manner. The METTL3-CONT7-YTHDF1 regulatory axis might be the potential target for the diagnosis and therapy of OS.

## Data Availability

The data used to support the findings of this study are available from the corresponding author upon reasonable request.
